# Pensando sobre diferentes lugares e papeis

**DOI:** 10.1590/2317-1782/20242024011pt

**Published:** 2024-06-14

**Authors:** Fernanda Dreux Miranda Fernandes

**Affiliations:** 1 Faculdade de Medicina, Universidade de São Paulo – USP - São Paulo (SP), Brasil.

Colonizar ou ser colonizado é a escolha necessária? Essas são as únicas alternativas?

Vamos restringir a argumentação para a Fonoaudiologia.

No que diz respeito à oferta de serviços, somos colonizados quando assumimos que a melhor forma de atender à população são as seções individuais, algumas vezes por semana. Também somos colonizados quando *importamos* métodos e modelos de avaliação e intervenção produzidos em outra língua e em contextos muito diferentes das realidades brasileiras.

Na ciência, somos colonizados quando reproduzimos modelos de pesquisas desenvolvidos e ajustados para contextos muito diferentes dos nossos. A necessidade de divulgar a ciência brasileira em inglês – mesmo pensando que essa é a “língua da ciência” – é mais um indício de colonização, assim como a busca por indexação em bases de dados internacionais. Não é o meu objetivo aqui discutir a necessidade de publicações “indexadas internacionalmente” para o reconhecimento dos pesquisadores brasileiros, mas acho que podemos pensar em o quanto isso coloca esse reconhecimento fora da nossa realidade.

Por outro lado, colonizamos quando trabalhamos com a noção de que dominamos um conhecimento e temos o dever (e a *autoridade*) de *informar* a população sobre parâmetros e técnicas que *dominamos*. Por exemplo, quando um fonoaudiólogo propõe uma ação de *prevenção*, em que pais e cuidadores recebem informações sobre o desenvolvimento da linguagem, esse profissional está transmitindo conhecimentos que podem ser tão diferentes das realidades das famílias e das dificuldades (ou questões) reconhecidas por elas, que o resultado é a falta de interesse ou de adesão ao programa de prevenção. Em ciência, colonizamos quando impomos a pesquisadores brasileiros parâmetros relacionados a *o que é ciência*, como se eles fossem indiscutíveis; quando consideramos que as pesquisas realizadas no Brasil devem ter as mesmas características, independentemente da realidade pesquisada.

Discussões sobre o *apagamento* de grupos minoritários (ou simplesmente menos poderosos) estão presentes na mídia e em atividades acadêmicas. Tem ficado cada vez mais claro como o hábito de descrever a realidade a partir da perspectiva do grupo dominante gera um empobrecimento para esse mesmo grupo. O desafio atual é o de ampliar o olhar e incluir o objeto de estudo na própria análise.

Não se trata de rejeitar todos os processos anteriores de construção. A Fonoaudiologia brasileira desenvolveu-se e forma consistente a partir da aplicação e da adaptação de conhecimentos produzidos em outras realidades. O desafio de publicar em outra língua e de apresentar o conhecimento a respeito da população brasileira de forma a gerar interesse a esse respeito em publicações relacionadas a outras línguas e realidades continua sendo relevante e gerou perspectivas diferentes nas diversas áreas da Fonoaudiologia. Por exemplo, fica claro que informações a respeito de audição, voz e sistema motor oral provavelmente encontrarão menos barreiras linguísticas do que aquelas relacionadas à linguagem, em que especificidades de uma certa língua podem não gerar interesse. Essa realidade tem mudado recentemente, provavelmente como resultado da mobilidade populacional e a necessidade de adaptar refugiados e migrantes em contextos diferentes. Algumas publicações a respeito de outras línguas – que não o inglês – tem sido mais frequentes e diversos periódicos internacionais. Entretanto, a maioria dessas publicações considera essa como a *segunda língua*. A construção de um volume de publicações em periódicos internacionais já coloca a Fonoaudiologia brasileira num lugar de destaque internacional.

O trabalho consistente, dedicado e rigoroso de editores científicos, revisores e autores, ao longo de décadas, possibilitou a indexação de diversos periódicos científicos da Fonoaudiologia brasileira em bases de dados internacionais e, sem dúvida, a indexação da CoDAS na Web of Sciences é uma vitória importantíssima.

Será, então, que não podemos pensar em ampliar nossos horizontes?

Discussões sobre uma prática profissional efetivamente inclusiva seguramente vão ter que levar em conta diferentes hábitos, crenças e costumes da população-alvo, possibilitando trocas de aprendizado entre quem oferece e quem recebe o serviço^(1, 2)^. Da mesma forma, quando consideramos um sistema universal de saúde, é fundamental pensar em alternativas eficazes para garantir os serviços de Fonoaudiologia a toda a população; e parece pouco provável que isso seja atingido no modelo de sessões individuais de intervenção. A tecnologia digital, a inteligência artificial e o letramento digital são temas que tem sido abordados de forma inconsistente em Fonoaudiologia; eles podem resultar em algumas soluções, mas também podem ampliar as dificuldades se não forem abordados de forma consciente e ética. Esses são alguns dos temas a serem considerados por quem aceitar o desafio de descolonizar a prática profissional em Fonoaudiologia.

Nesse contexto, o papel da CoDAS na discussão de alterativas para a *descolonização* da Fonoaudiologia é fundamental. Não se trata de ignorar as normas internacionais para publicações, nem de colocar em risco a indexação conquistada. Mas estou convidando editores, revisores e autores a pensarem sobre alternativas viáveis para isso. Seria possível criar uma sessão “*Fora da Caixa*” para artigos que não se encaixem em normas rigorosas de publicação? Um ponto fundamental seria identificar *porque* não se encaixam ou se esse exercício causaria a perda de dados relevantes. Outra alternativa seria a identificação de *mentores* que pudessem trabalhar com os autores de um manuscrito com potencial mas que dificilmente seriam tornados adequados para publicação através do sistema de revisão tradicional^(3)^. A história pessoal de autores e/ou grupos de pesquisa podem ser um tema a ser abordado nessa descolonização. Autores com necessidades especiais podem oferecer perspectivas enriquecedoras para a abordagem de necessidades específicas. Autores de diferentes grupos sociais (como povos originários, migrantes ou refugiados) podem contribuir para a discussão de estratégias de inclusão.

Não tenho dúvidas de que há inúmeros temas, possibilidade e dificuldades para o início de um processo de *descolonização*. Mas somos fonoaudiólogos e sabemos que a melhor forma de começar um processo é pensar a respeito dele. Esse é o meu convite.
